# Spectroscopic analysis of nanosized Zn(Ag, Ni)O systems and observation of superparamagnetism at low temperature

**DOI:** 10.1039/d4na00077c

**Published:** 2024-06-14

**Authors:** Kamakhya Prakash Misra, Saikat Chattopadhyay, Atul Bandyopadhyay, Albin Antony, Ashok Rao, P. Poornesh, J. Jedryka, K. Ozga, B. Kucharska, Yanting Yin, Gunther Andersson, Arunava Agarwala, Yung-Kang Kuo

**Affiliations:** a Department of Physics, School of Basic Sciences, Manipal University Jaipur Jaipur Rajasthan 303007 India saikat.chattopadhyay@jaipur.manipal.edu the_saikat@yahoo.com; b Department of Physics, University of Gour Banga Malda West Bengal 732103 India; c Department of Surface and Plasma Science, Faculty of Mathematics and Physics, Charles University V Holesovickach 2 180 00 Praha 8 Czech Republic; d Department of Physics, Manipal Institute of Technology, Manipal Academy of Higher Education Manipal Karnataka 576104 India poornesh.p@manipal.edu poorneshp@gmail.com; e Faculty of Electrical Engineering, Czestochowa University of Technology Armii Krajowej 17 PL-42-201 Czestochowa Poland; f Faculty of Production Engineering and Materials Technology, Czestochowa University of Technology Armii Krajowej 19 Częstochowa 42-200 Poland; g Future Industries Institute, University of South Australia, Mawson Lakes Campus SA 5095 Australia; h College of Science and Engineering, Flinders University Adelaide SA 5001 Australia; i Department of Chemistry, Malda College Malda West Bengal 732101 India; j Department of Physics, National Dong Hwa University Hualien 97401 Taiwan

## Abstract

To understand the impact of binary doping in ZnO, nanosized Zn(Ag, Ni)O systems were synthesized by the sol–gel method. The amount of Ag was fixed at 2 at%, and that of Ni was varied from 1 to 15 at%. Ni incorporation equal to or beyond 3 at% gave rise to the development of the NiO phase. The presence of Ag and Ni did not have much influence on the lattice constants of ZnO. However, a larger addition of Ni impacted the unit cell of NiO, as indicated by the reduction of the lattice constant of NiO. The increase in NiO and Ag contents in ZnO reduced the second and third harmonic intensities under non-linear investigations. X-ray photoelectron spectroscopy analysis indicated that initial Ni addition varied randomly along with Ag, and it stabilized itself at higher concentration. Field emission scanning electron microscopy revealed that interlinked particles and chains with tamarind shapes were formed, closely matching the rod-like structures under high resolution. Ag and Ni addition altered the structures slightly and randomly till 5 at% Ni; thereafter they deviated from the particle shape to flat disc-shapes. Interestingly, the magnetic response of the sample was determined by the NiO phase, and the effect of Ni and Ag substitution in the ZnO host matrix was almost irrelevant at low temperatures toward magnetic contribution. Weak ferromagnetism at low temperatures (≤50 K) with superparamagnetic-like behavior (cusp in ZFC magnetization) was observed in all the samples. This could be attributed to the finite nano-size effect and uncompensated spins at the surface of the particle.

## Abstract 2

To understand the impact of binary doping in ZnO, nanosized Zn(Ag, Ni)O systems were synthesized by the sol–gel method.

## Graphical abstract

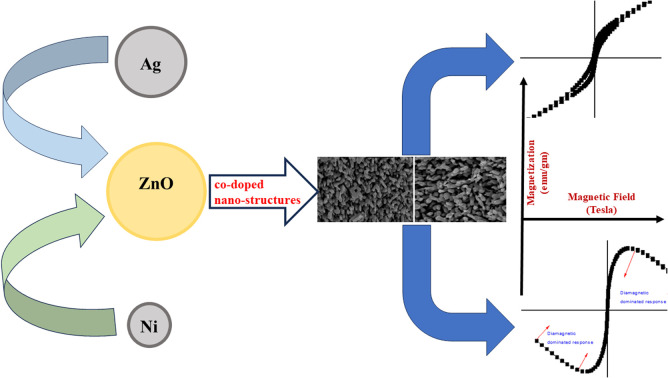

## 1. Introduction

Among various semiconductor nanomaterials, ZnO nanoparticles (NPs) are some of the most promising materials for various electronic and optoelectronic applications due to their wide band gap energy and large exciton binding energy at room temperature. In addition, ZnO NPs have been applied in numerous fields, including diluted magnetic semiconductors, optical devices, cosmetics, photocatalysis, *etc.*^1–3^

Due to their versatility, there is an increasing demand for suitable modifications in ZnO NPs to achieve improved and efficient features. A variety of transition and non-transition metals have been doped into ZnO to improve its optical properties. Ca, Ni, Sr, Ag, Ce, Eu, Co, *etc.*, are popular examples of dopants incorporated into ZnO for gaining favorable luminescence, a tuned band gap, and improved ferromagnetism.^4–9^ The enhancement of the photocatalytic activity of Co-doped ZnO nano-disks/-rods produced by wet chemical techniques proved that a large facet area and acceptable band gap favour superior photocatalytic action in the grown nanostructures.^10–13^ ZnO thin films exhibit better electrical properties along with an improvement in crystalline quality when there is the formation of nanorods or they are doped with Cu.^13^ Particularly, Ni offers fine-tuning of optical properties by inducing impurity levels between the valence band and the conduction band, promoting visible light absorption and reducing charge carrier recombination.^1,5^ The indirect interaction between oxygen vacancies and transition metal dopant ions forms magnetic polarons, which overlap to generate room-temperature ferromagnetism.^2^ Recently, co-doping has drawn significant attention as it offers better optoelectronic and magnetic features as compared to single metal ion doping in ZnO.

Multiple combinations have been reported that have demonstrated superior properties. Ag and Fe were used as co-dopants in a specific molar ratio in ZnO, and the resultant materials demonstrated excellent photocatalytic activity.^14^ Er and Dy co-doped ZnO systems also recently demonstrated superior photocatalytic activity.^15^ Zr–Ag–ZnO was found to be suitable for the degradation of RR120 dye.^16^ Sol–gel synthesized Y and Al co-doped ZnO exhibited excellent photocatalytic activity.^17^ Hence, it is pertinent to claim that co-doping has significance in improving the photocatalytic behavior of ZnO. A Ga and Al co-doped ZnO system was analyzed for diluted magnetic semiconductors.^18^ Recently, ferromagnetic ordering *via* bound magnetic polarons was observed in Al and Ce co-doped ZnO.^19^ However, there is no established consensus on which of the two transition metals should be co-doped into ZnO for gaining favorable magnetic behavior. Some efforts are visible in the literature when Ni and Ag together have shown a better reduction of nitro-compounds, but no spectroscopic and magnetic analysis has been done. Ni is generally found to reduce the charge carrier recombination by imparting the trap states, and Ag is a well-known electron donor; hence it also reduces the recombination of electrons.^1^ Therefore, it is expected that reduced carrier recombination will lead to the formation of bound magnetic polarons, which eventually will influence the magnetic behavior of ZnO.

Numerous techniques have been used to produce ZnO NPs/films, including hydrothermal, electrochemical, chemical vapour, thermal, combustion, chemical–thermal synthesis, anodization, sol–gel co-precipitation, and electrophoretic deposition. Most of them are costly and sophisticated. One of the most straightforward processes is sol–gel, which is economical, effective, and perfect for the development of doped ZnO nanoparticles.^4^ The other added advantages of sol–gel processing, particularly in the synthesis of oxide-based materials, are mild reaction conditions, low processing temperatures, ease of purification of molecular or soluble precursors and the easy modification of the chemical composition.

There are limited original research contributions towards the understanding of wide band gap semiconductor nanoparticles doped with two different elements simultaneously. Hence, with an objective to understand the impact of binary doping in ZnO, we herein report the synthesis of Ni and Ag co-doped ZnO by a sol–gel co-precipitation method. The underlying mechanism of how two dopants (Ni and Ag) can influence the structures, surface, and magnetic behaviour, is thoroughly analyzed *via* basic characterization techniques, such as XRD, XPS, and FESEM. Further the results obtained are corroborated with the overall performance of the synthesized material. The analysis of its potential as a harmonic generator is also included in the study. A thorough understanding of the magnetic properties of such a binary doped Zn(Ag, Ni)O system is also presented.

## 2. Experimental

### 2.1 Synthesis

An aqueous solution of PEG-400 (15 g PEG in 185 mL distilled water) was taken into a 1000 mL two-neck round-bottomed flask. Aqueous solutions of Zn(CH_3_COO)_2_·2H_2_O (15 mmol) and various mol% of dopant (Ni in the form of Ni(NO_3_)_2_·6H_2_O) and 2 mol% of co-dopant (Ag in the form of AgNO_3_) were prepared in 200 mL distilled water (solution A). Another aqueous solution of (NH_4_)_2_CO_3_ (12 mmol) in 200 mL distilled water was prepared (solution B). Solutions A and B were taken in two separate dropping funnels and added dropwise simultaneously into the round-bottomed flask with vigorous stirring. With the complete addition of solution B, the pH of the solution changed from almost neutral to more than 9. After the completion of addition, the resultant solution was further stirred for 2 h at room temperature, and the formation of precipitate was observed. The precipitate was filtered and washed with deionized water, followed by anhydrous ethanol, and dried at room temperature for 12 h. Finally, the precipitate was calcined in a furnace at 500 °C for 3 h. The amount of Ag in all the doped samples was kept at 2 at%, and the amount of Ni in all co-doped samples was varied as 1, 3, 5, 7, 10, 12, and 15 at%. The sample description and depiction are given below (Table 1).

**Table 1:** Sample Description

Sample designation	Sample content variation (two presentation styles)	
S1	ZnO	ZnO
S2	Ag_2__Ni_0__ZnO	ZnO/Ag
S3	Ag_2__Ni_1__ZnO	ZnO/Ag/1% NiO
S4	Ag_2__Ni_3__ZnO	ZnO/Ag/3% NiO
S5	Ag_2__Ni_5__ZnO	ZnO/Ag/5% NiO
S6	Ag_2__Ni_7__ZnO	ZnO/Ag/7% NiO
S7	Ag_2__Ni_10__ZnO	ZnO/Ag/10% NiO
S8	Ag_2__Ni_12__ZnO	ZnO/Ag/12% NiO
S9	Ag_2__Ni_15__ZnO	ZnO/Ag/15% NiO

### 2.2 Characterization

The prepared samples were characterized through X-ray diffraction (XRD) (Miniflex 600, Rigaku, Japan) and X-ray photoelectron spectroscopy (XPS) for their structural alterations by doping and co-doping. The details of the XPS instrument are described in ref. 20. Field emission scanning electron microscopy (FESEM) (JEOL-7610F, Japan) was used to judge the surface morphology. The second harmonic generation (SHG) and third harmonic generation (THG) studies were performed using a similar experimental set-up to that described in ref. 21. For the assessment of magnetic properties, magnetization *versus* field (*M*–*H*) curves were recorded using a vibration sample magnetometer (VSM) (PPMS, Quantum Design, USA).

## 3. Results and discussion

### 3.1 XRD

**Fig. 1:**
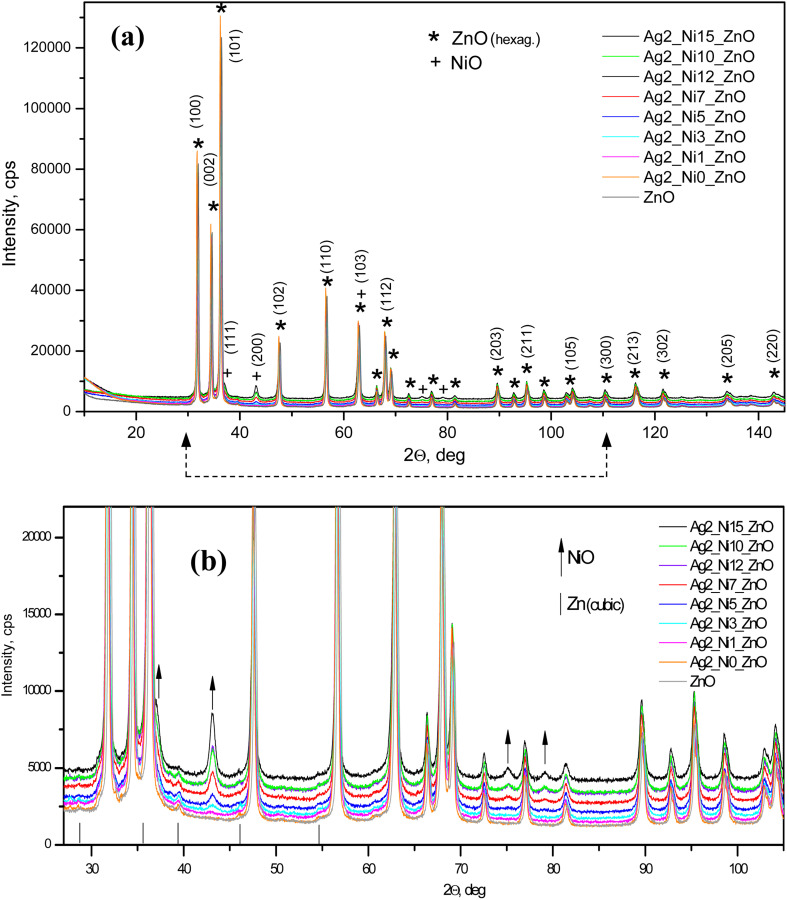
X-ray diffractograms of the (a) Ag-doped and (b) Ni co-doped ZnO NPs.

**Fig. 2:**
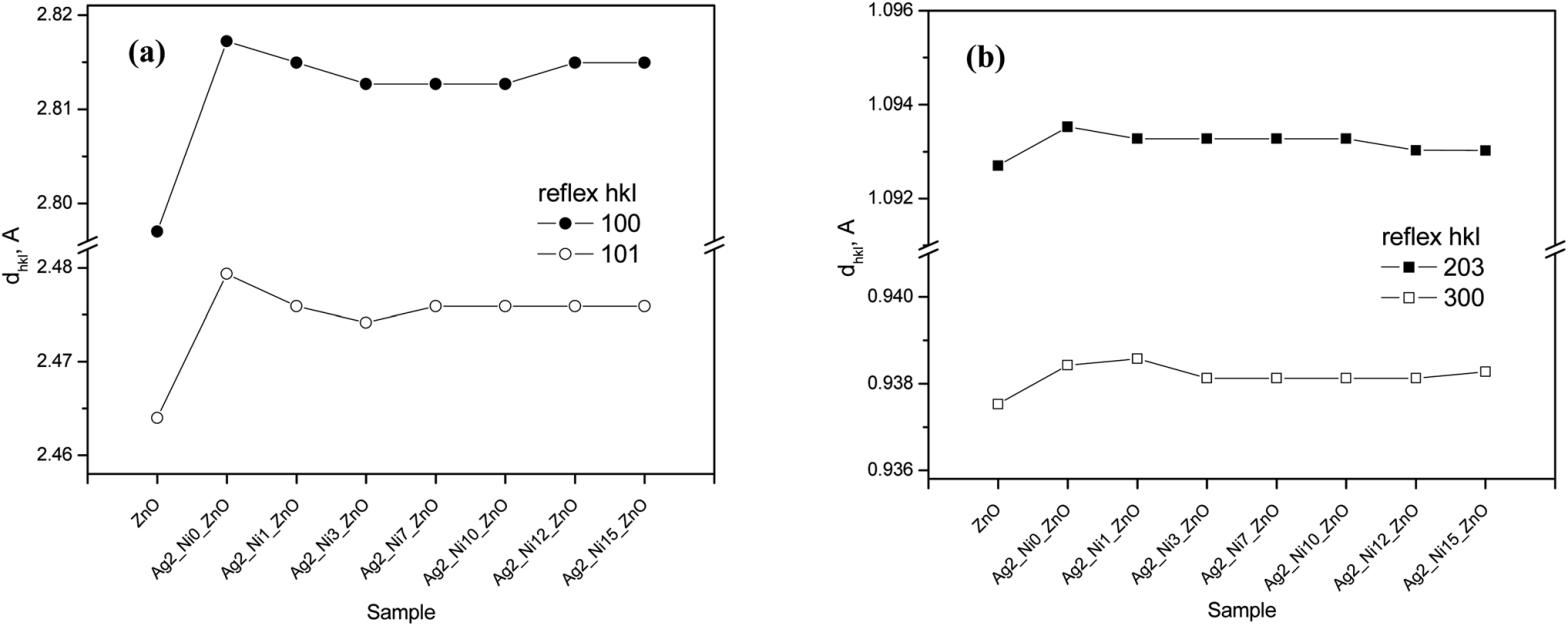
Influence of dopants on principal inter-plane distances *d*_*hkl*_ for the structure of (a) Ag-doped and (b) Ni co-doped ZnO NPs.

Fig. 1(a) shows the XRD patterns of all the samples. All the characteristic peaks corresponding to the hexagonal wurtzite phase (ICDD Card No. 89-1397) of ZnO are visible.^21^ Any new phase does not appear up to S2, which is a 1 at% Ag doped ZnO sample. From samples S3 to S9, the amount of Ni increases as described in the synthesis section. Accordingly, the magnified peaks (Fig. 1(b)) reveal the formation of NiO from S3 to S9. The peak intensities corresponding to NiO monotonically increase as the amount of Ni increases in the ZnO matrix. The upward arrows are marked to correspond to NiO peaks in Fig. 1(b). The incorporation of Ag does not show any new phases; however, the diffraction angles shift to lower angles, thereby increasing the interplanar distance (*d*). The shift in diffraction angles corresponding to (100), (002), and (101) peaks is larger, and it is smaller for other peaks. Accordingly, we notice a greater increase in *d* (almost by 2 Å) for (100) and (101) and a smaller increase in other peaks like (203) and (200), as shown in Fig. 2.

**Fig. 3:**
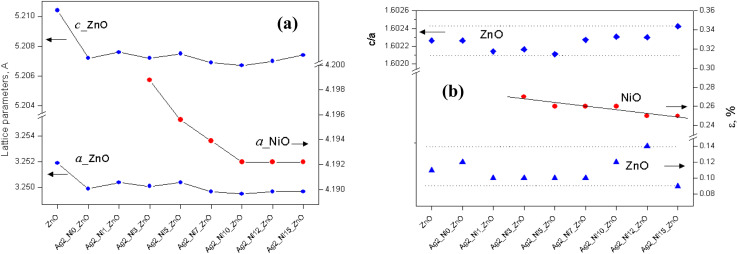
Influence of dopants on (a) the lattice parameters of phases and (b) the *c*/*a* ratio for ZnO and lattice disturbance for the structures of ZnO and NiO.

The enhancement of *d* suggests that Ag replaces Zn in ZnO. This replacement preferentially takes place along the densest planes. Despite an enhancement of the interplanar spacing, the lattice constants (*a* and *c*) reduce (see Fig. 3(a)). This reduction is prominent only with Ag doping. Ni incorporation does not cause any major impact on *a* and *c*. The values of *a* and *c* for the ZnO samples with higher amounts of Ni (S3 to S9) fluctuate randomly about 5.207 Å and 3.250 Å, respectively.

However, the lattice constant *a*_Ni_ of NiO decreases from S4 to S9 and thereafter achieves saturation. Thus, it can be concluded that more Ni incorporation compresses the NiO unit cell, thereby reducing *a*_Ni_. An important parameter related to the hexagonal crystal structure, *i.e.*, *a*/*c* ratio, remains unaffected by Ag doping (S2). Then the addition of Ni reduces it slightly up to S5, and it increases (Fig. 3(b)). The lattice deviations for ZnO and NiO are also shown in Fig. 3(b). It is found that the percentage lattice deviation for NiO goes down monotonously from 0.27% to 0.25% from S4 to S9. However, the percentage lattice deviation of ZnO does not depend on Ni content. Doping a semiconductor with foreign elements is expected to cause strain in the samples because of changes in lattice constants. The deviation of lattice constants compared to that of bulk ZnO gives the amount of strain available in the samples. The strain values calculated for all the samples are given in Table 2. The negative values of strain demonstrate its compressive nature. There is no definite trend of variation as is visible from the strain values.

**Table 2:** Calculated strain (along the 100 and 101 planes) and dislocation density along the strongest crystallographic planes

Samples	Strain		Dislocation density (*δ*) × 10^14^ m^−2^
	Along (100)	Along (101)	Along the strongest peak
S1	−0.01	−0.007	5.863
S2	−0.003	−0.001	4.377
S3	−0.003	−0.002	8.210
S4	−0.004	−0.003	7.631
S5	−0.004	−0.002	7.631
S6	−0.004	−0.002	7.980
S7	−0.004	−0.002	8.353
S8	−0.003	−0.002	7.935
S9	−0.01	−0.007	8.702

**Fig. 4:**
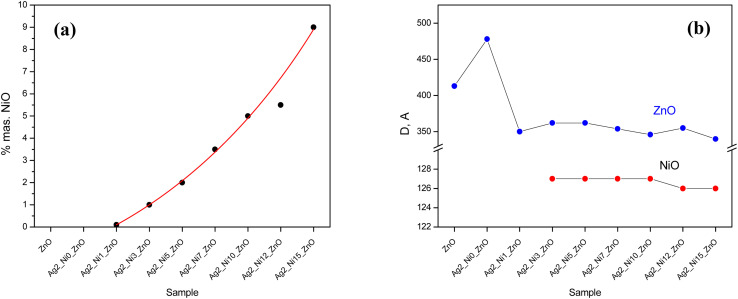
Influence of the dopant and co-dopant on (a) the participation of NiO and (b) the sizes of ZnO and NiO for the samples.

We also identified the mass percentage of NiO, and the results are shown in Fig. 4(a) with the expected trend of systematic increment. The maximum content of NiO is 9%, corresponding to the sample with 15 at% Ni and 2 at% Ag in ZnO.

The oxides ZnO and NiO possess different crystalline sizes; the variation is shown in Fig. 4(b). The averaged crystallite size of ZnO increases with Ag incorporation (S2). When Ni is introduced along with Ag (S3), it drops suddenly and thereafter remains almost constant, close to a value of 353 Å. The averaged crystallite size of NiO lying close to 127 Å remains constant up to S7; after that, it drops slightly (S8 to S9). The addition of dopants produces dislocations and the dislocation density in the case of ZnO is the inverse square of the crystallite size. The calculated values of dislocation density for all the samples along the strongest crystal planes are listed in Table 2.

### 3.2 XPS

**Fig. 5:**
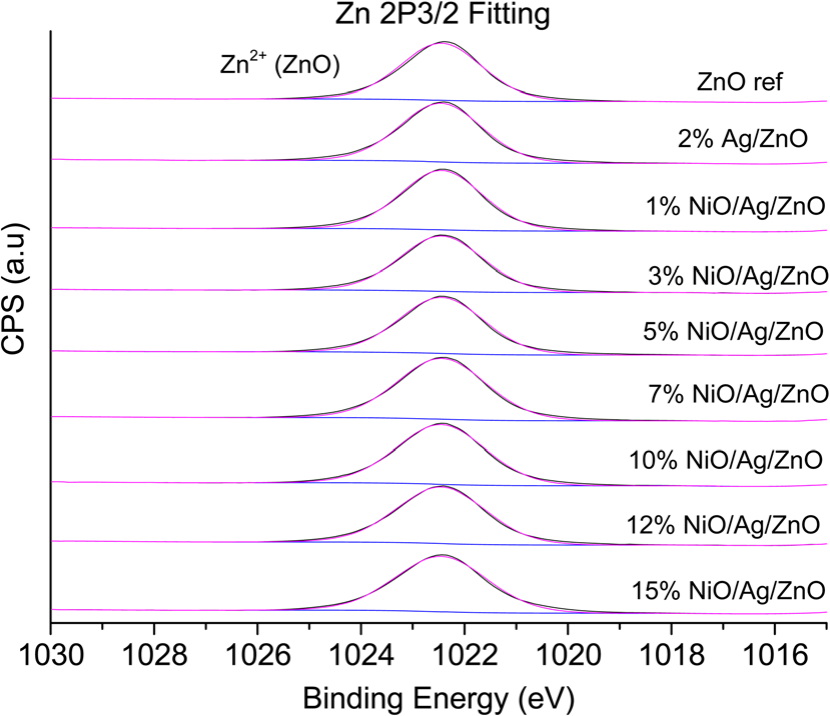
Zn 2p_3/2_ fitting spectrum obtained from the XPS spectra of Ag-doped and Ni co-doped ZnO NPs.

**Fig. 6:**
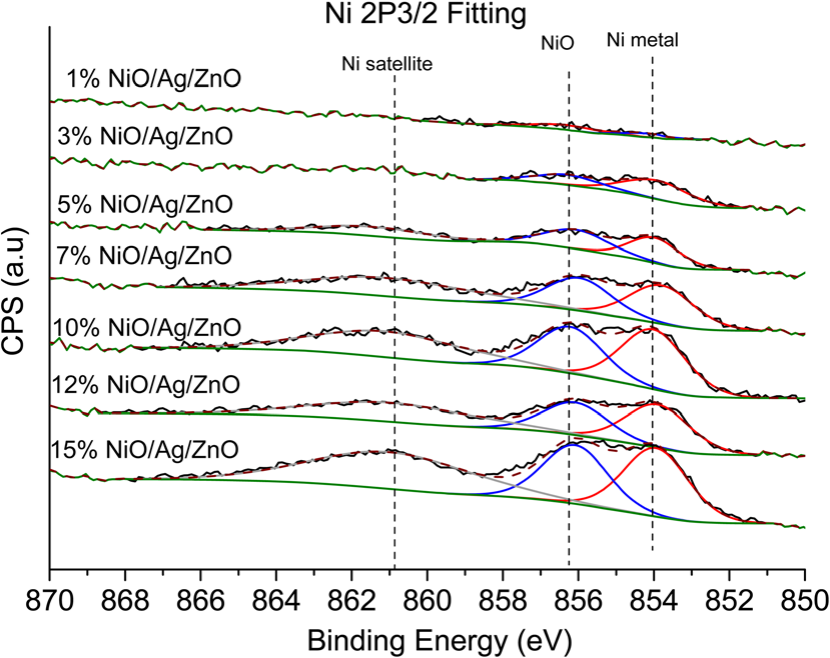
Ni 2p_3/2_ spectrum obtained from the XPS spectra of Ag-doped and Ni co-doped ZnO NPs.

XPS is a useful technique to investigate the valence states of the elements present in the material.^22–24^ The XPS spectra of all the samples are recorded, and Zn and Ni peaks are detected, as presented in Fig. 5 and 6. The Zn 2p peak shows a symmetrical Gaussian shape, discarding the presence of multicomponent Zn species. The peak at 1022.5 ± 0.2 eV, attributed to Zn 2p_3/2_, is visible in the S1 sample, as seen in Fig. 5. A slight shift of the peak towards higher binding energy after Ag doping (S2) could potentially be due to the substitution of Zn by Ag. However, Ni co-doping (S3–S9) does not cause any shift of the peak, indicating the absence of substitution reactions by Ni in the matrix of ZnO.^22–24^ A similar conclusion was obtained from the XRD analysis. To understand the distribution of Ni in ZnO, the related peaks are shown in Fig. 6. Ni core level peaks corresponding to Ni 2p_3/2_ are observed at 854 ± 0.2 eV, whereas the corresponding satellite peaks are seen at 859.8 ± 0.2 eV. The local structure of Ni atoms governs the peak position. Actually, the peak corresponding to 854 ± 0.2 eV is due to the metallic Ni atom. Another peak at 856.3 ± 0.2 eV corresponds to NiO. A systematic rise of the peaks from S3 to S9 indicates an increase in the Ni-related species in the ZnO matrix. Such an observation is in accordance with the XRD results.

**Fig. 7:**
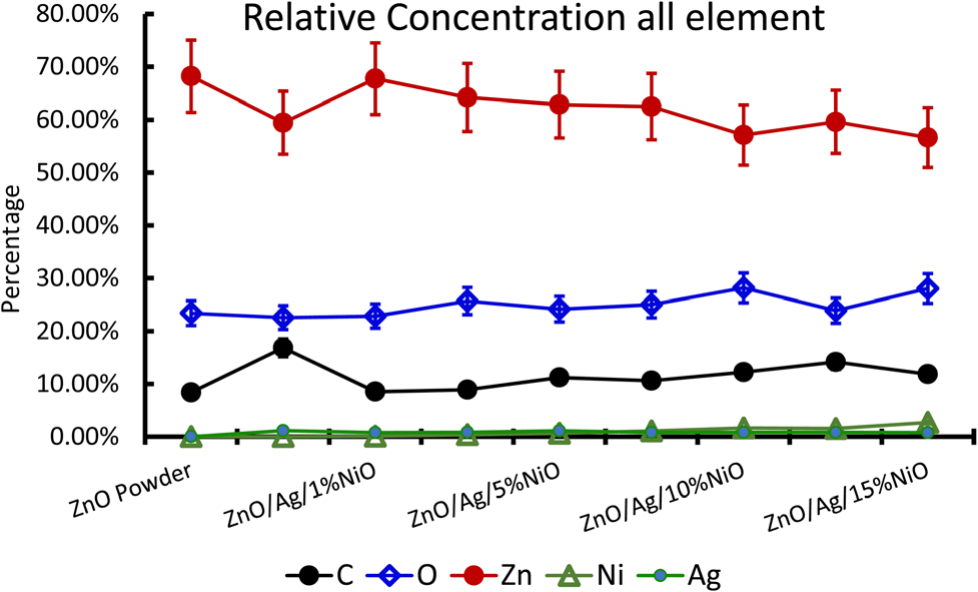
Relative concentration of all the participating elements in Ag-doped and Ni co-doped ZnO NPs.

**Fig. 8:**
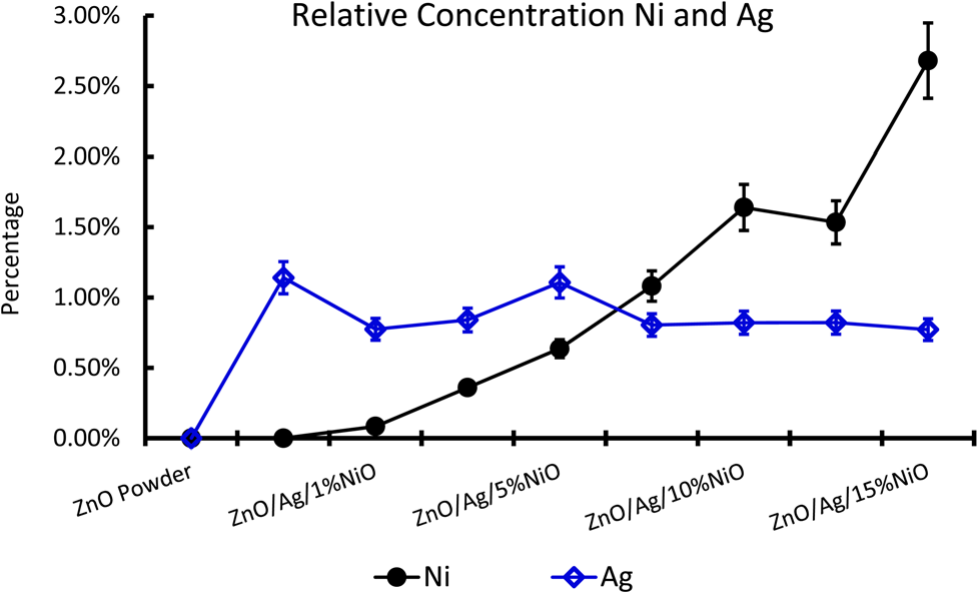
Relative concentration of Ag and Ni in Ag-doped and Ni co-doped ZnO NPs.

**Fig. 9:**
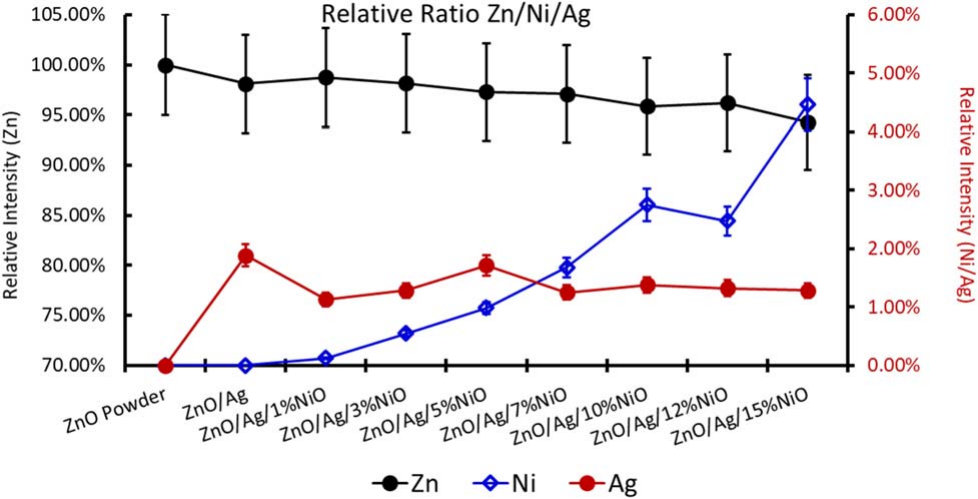
Relative ratio of Zn, Ni, and Ag in Ag-doped and Ni co-doped ZnO NPs.

**Fig. 10:**
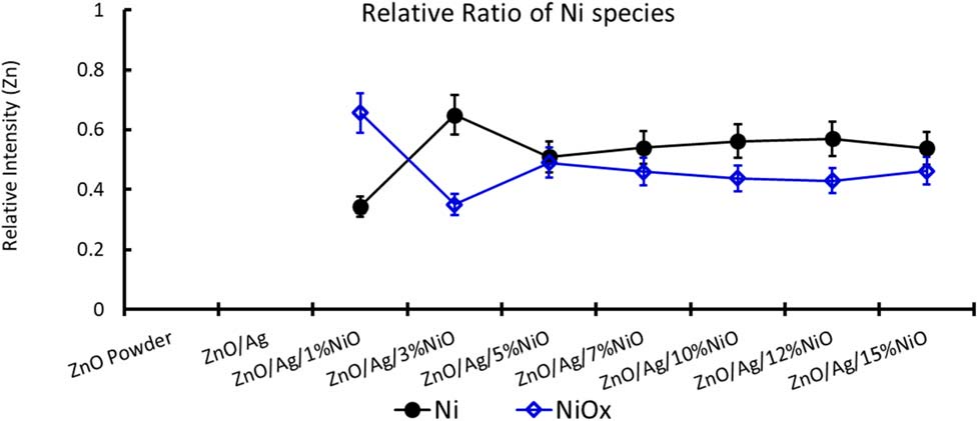
Relative ratio of Ni species in Ag-doped and Ni co-doped ZnO NPs. Note that on the multi-*y*-axis the element is labelled individually.

For further clarity on elemental species present in the samples, the relative concentrations of different components are presented in Fig. 7. As the concentration of Ag is very low in all doped samples (S2–S9), their oxidation state cannot be identified. The significant and appropriate percentages of all other species like Zn, O, and Ni are clearly visible in Fig. 7. As the samples have compositional variation in terms of dopant (Ag) and co-dopant (Ni), the relative concentration variation of Ni and Ag is shown in Fig. 8. The variation of the elements with samples closely follows the expected curves with Ni increasing as the doping amount increases. For comparison, the experimentally selected composition values of Ni and Ag are shown in the inset of Fig. 8. Zn, Ag, and Ni are the major entities expected to participate in the substitution reaction process. Hence, their relative ratio is presented in Fig. 9. All three entities follow the expected trend in line with experimentally selected amounts. Ni exists mainly in two forms, *i.e.*, Ni and NiO. The relative intensities of both Ni species are shown in Fig. 10. Both of them are available within the samples with almost the same ratio. Initial doping (S3–S4) causes the exchange of these species with each other, and they follow nearly the same curves. This indicates that initial Ni addition remains unstable, stabilizing at higher concentrations in ZnO.

### 3.3 FESEM

**Fig. 11:**
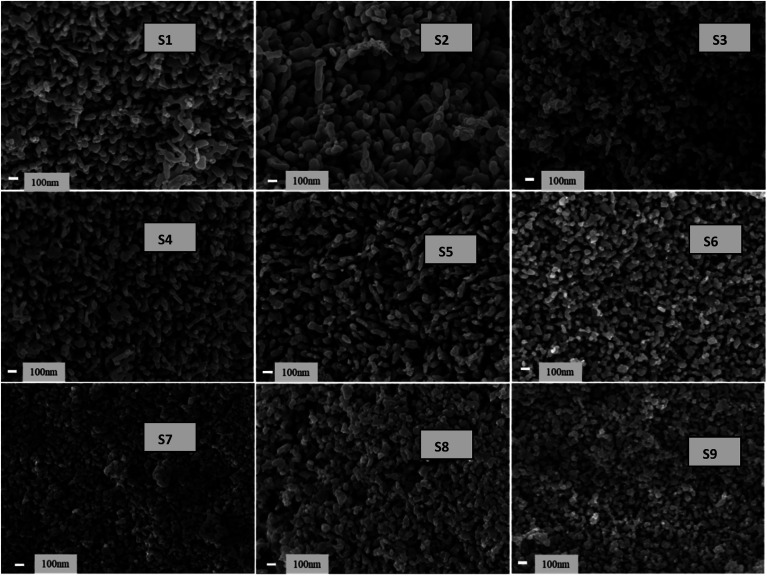
FESEM images of Ag-doped and Ni co-doped ZnO NPs at low magnification.

**Fig. 12:**
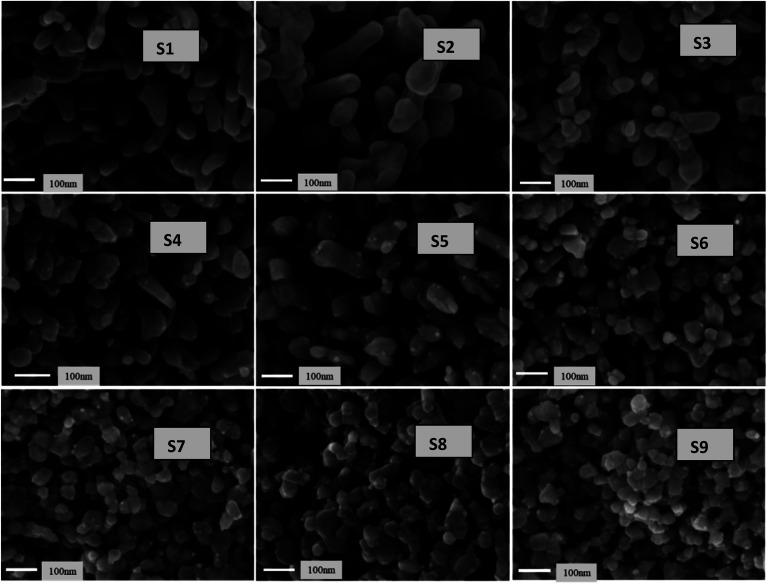
FESEM images of Ag-doped and Ni co-doped ZnO NPs at high magnification.

Fig. 11 presents the low-magnification FESEM images of all samples. The images reveal interlinked particle chains that have tamarind shapes. The tamarind shapes are more prominent with Ag doping in ZnO (S2). When Ni is incorporated, the morphology deteriorates initially to a random distribution of particles (S3), and then it again adjusts towards chained particles, taking a rod-like outgrowth for the S4 to S6 samples. A higher addition of Ni in Ag-doped ZnO starts forming clusters, and there is no definite morphological variation. However, the particles remain visible at the highest level of Ni doping. Fig. 12 shows the high-magnification images, where randomly distributed particles of mixed shapes and sizes are visible.

### 3.4 Second and third harmonic studies

**Fig. 13:**
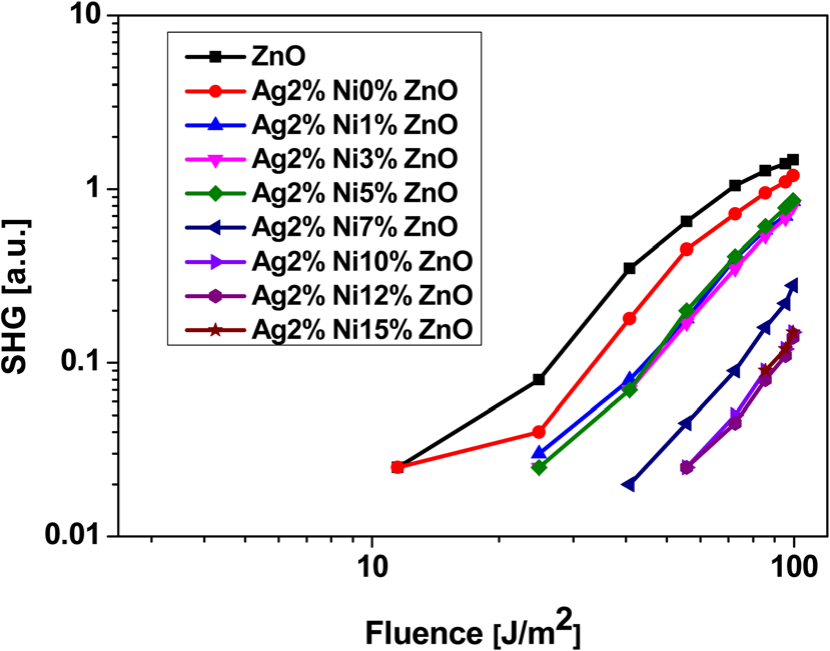
Laser-stimulated SHG efficiency of Ag-doped and Ni co-doped ZnO nanoparticles *versus* the fundamental laser energy.

**Fig. 14:**
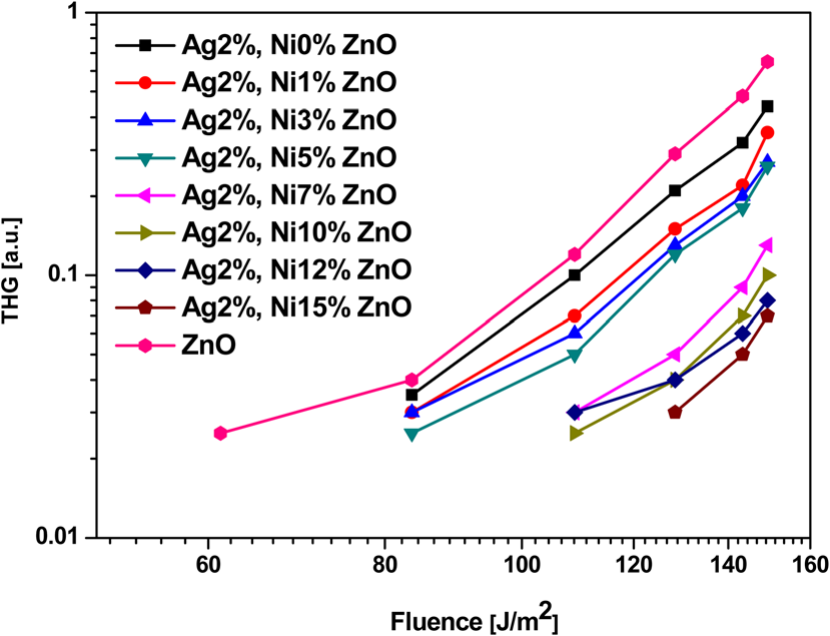
Laser-stimulated THG spectra of Ag-doped and Ni co-doped ZnO NPs.

Second and third harmonic generation (SHG and THG) occur as a result of nonlinear interaction between the material and the incident radiation. In the case of ZnO, each Zn ion is surrounded by a tetrahedron of four O ions and *vice versa* in the hexagonal wurtzite phase. This tetrahedral coordination results in a non-centrosymmetric crystal structure, which makes it a promising candidate to observe SHG under ambient conditions.^21^ The dependence of the signal intensity of SHG and THG *versus* that of the fundamental beam is shown respectively in Fig. 13 and 14. The variation of SHG signal intensity shows that the incorporation of Ag and Ni reduces the signal strength but it saturates for S5 (Ag 2 at% and Ni 5 at% doping). A drastic drop is observed for further addition of Ni in Ag-doped ZnO. In ZnO nanostructures and thin films, there are numerous factors that affect the SHG signal intensity, *e.g.*, the contribution from crystalline quality, surface defects, doping level, aspect ratio, *etc.*^25^ All these factors are significantly affected by Ag doping and Ni co-doping. Hence, a drop in SHG signal intensity is expected. A similar variation of THG signal intensity is observed. The decay of the signal with doping is impacted in the same way as in SHG. THG is generally observed in symmetry-allowed materials, while SHG is available with a lack of inversion symmetry.^25^ In the present study, a co-doping strategy with Ag and Ni has been adopted; hence the compensation of the influence of both dopants may result in the decay of THG.

### 3.5 Magnetic study

**Fig. 15:**
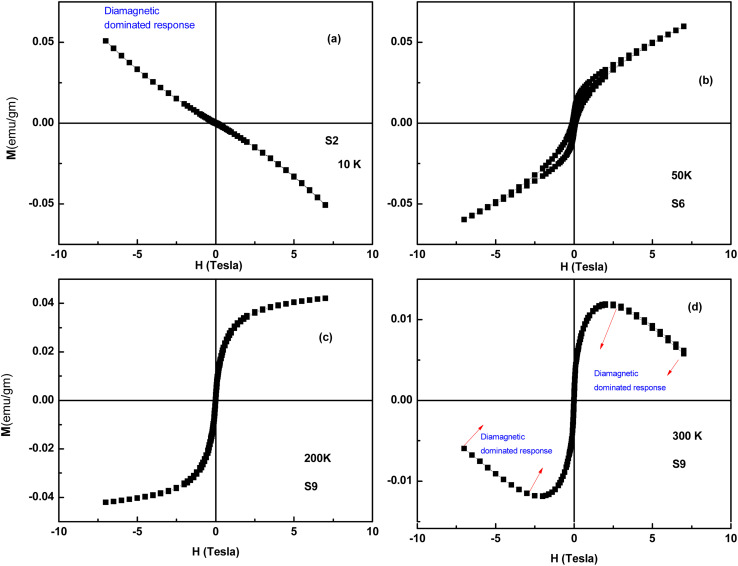
*M*–*H* loops of Ag-doped and Ni co-doped ZnO NPs. (a) S2 (ZnO/Ag) at 10 K, (b) S6 (ZnO/Ag/7% NiO) at 50 K, (c) S9 (ZnO/Ag/15% NiO) at 200 K and (d) S9 (ZnO/Ag/15% NiO) at 300 K.

VSM is a versatile technique for measuring the magnetic moment of a sample when it is vibrated perpendicularly to a uniform magnetizing field. VSM measures the magnetic moment of the entire sample and magnetization *versus* field (*M*–*H*) curves, typically called hysteresis loops, are plotted. Here, we recorded *M*–*H* loops at different temperatures (300, 200, 50, 20, and 10 K) and *M*–*T* data under field-cooled (FC) and zero-field conditions (ZFC) (200 Oe) for all the samples. The *M*–*H* loops of selected samples are shown in Fig. 15. The samples S3 to S9 exhibit a distinct hysteresis loop up to 20 K, whereas the S1 and S2 samples are diamagnetic in nature. It is to be noted that only the S3 and S4 samples exhibit a hysteresis loop up to 20 K, whereas the samples S5 to S8 exhibit a hysteresis loop up to 50 K. Among all the samples, S9 has a better magnetic response and a distinct hysteresis loop up to 200 K. The *M*–*H* curve at 300 K of S9 indicates that the sample is a mixed-phase material, where ferromagnetic phases coexist with strong diamagnetic phases. The magnetic data, along with XRD data, suggest that hysteretic behavior arises from the NiO phase, and diamagnetic response arises from ZnO or doped ZnO phases. Because of the ZnO host and the lack of the NiO phase, the S1 and S2 samples are diamagnetic. The S9 samples exhibit a distinct hysteresis loop at the highest temperature compared to all the samples due to the highest weight percentage of the NiO phase. Interestingly, none of the hysteresis curves reaches saturation in spite of the high applied field (∼6*T*). In the nano-regime, a larger number of particles reside on the surface compared to the bulk. Therefore, exchange bonds are broken on the particle surface, which gives rise to a large number of uncompensated spins at the surface and contributes to a linear response in the hysteresis loop.^26^

**Fig. 16:**
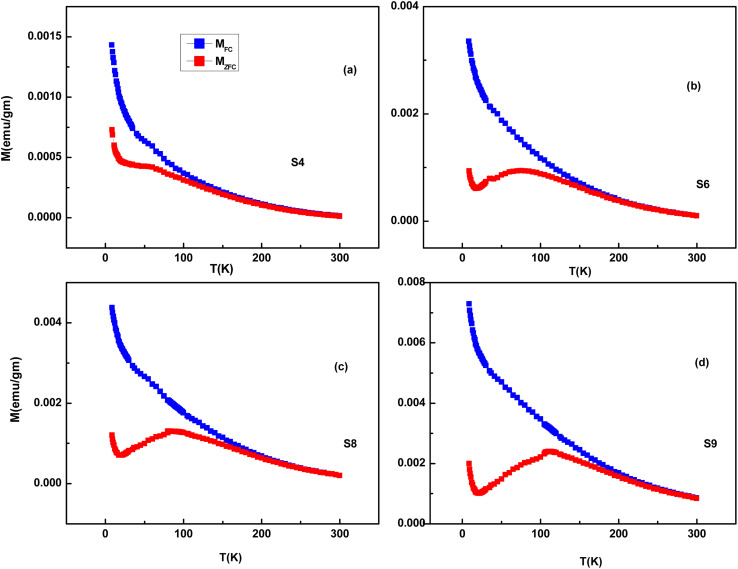
ZFC and FC *M*–*T* curves of Ag-doped and Ni co-doped ZnO NPs. (a) S4 (ZnO/Ag/3% NiO), (b) S6 (ZnO/Ag/7% NiO), (c) S8 (ZnO/Ag/12% NiO) and (d) S9 (ZnO/Ag/15% NiO).

The bifurcation between the FC and ZFC curves is observed for the S3–S9 samples, indicating magnetic ordering or super-paramagnetism within the samples. In sufficiently small nanoparticles, magnetization can randomly flip the direction under the influence of temperature, which is called super-paramagnetism. We have shown the *M*–*T* data of four samples in Fig. 16. The ZFC-FC plots are prominent despite the presence of a diamagnetic phase because the data were taken at a low field (∼200 Oe), where ferromagnetic/super-paramagnetic response is much higher than that in a diamagnetic phase. The ZFC curves of the S4 to S9 samples consist of a broad cusp rather than a sharp point, an indication of the presence of super-paramagnetic particles with a wide size distribution. The sharp maxima are the feature of a monodispersed particle. Still, for systems with a size distribution, the blocking temperature is distributed due to variation in the particle size, and cusp-like features were observed.^27,28^ Another reason for such a shape of the ZFC curve may be the distribution of point defects over the whole particle region.^29,30^ We don't observe any spin freezing in the FC/ZFC curve down to the lowest temperature. This may be because some of the surface spins are not exchange-coupled with the AFM core, which results in a paramagnetic contribution to total magnetization.^31^ On the other hand, E. De Biasi *et al.* explained that the sharp rise at low temperatures is due to the breaking of a large number of exchange bonds on the particle surface, driving the spins to a strongly frustrated state.^32^ As some fractions of Ni ions are properly doped in ZnO hosts, their contribution to paramagnetism cannot be ruled out.

It is well known that NiO in bulk form is antiferromagnetic with Néel temperature at ∼523 K. Usually, antiferromagnetic materials have no coercivity, and the *M*–*H* curve is almost linear. Below the blocking temperature, the competition between the Zeeman energy and the magnetic anisotropy energy results in potential barriers for the uncompensated magnetic moment and causes hysteretic behavior in single-domain particles. Therefore, based on the above discussion, the robust deviation of our samples can be described by the core–shell model. Thus, each nanoparticle has an AFM core with a surface spin subsystem. Therefore, the magnetization can be written as*M*_Total_ = *M*_FM_ + *M*_PM_ + *M*_AFM_

**Fig. 17:**
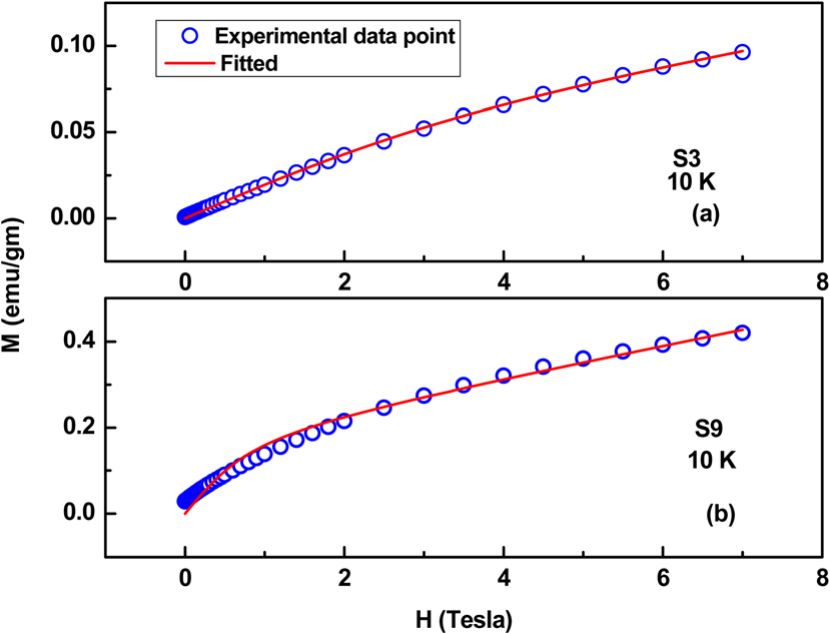
Representative fitting curves (*M*–*H*) based on a core–shell model. (a) S3 (ZnO/Ag/1% NiO) and (b) S9 (ZnO/Ag/15% NiO).

Assuming that both anti-ferromagnetic and paramagnetic responses have a linear relationship with the applied field*M*_PM_ + *M*_AFM_ = (*χ*_PM_ + *χ*_AFM_)*H* + (*χ*_Total_)*H*whereas,
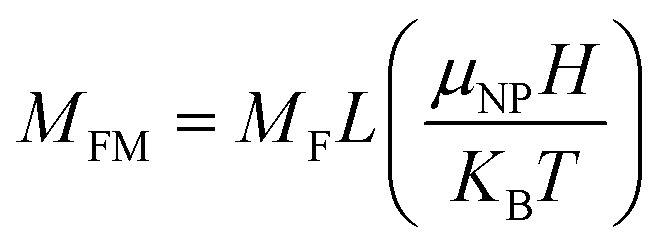


Here, *μ*_NP_ is the magnetic moment corresponding to each NP, and *L* stands for the Langevin function.^31^ The S3 to S9 samples exhibit a hysteresis loop at 10 K, and the *M*–*H* curves at 10 K are well fitted to the above core–shell model, which substantiates the applicability of this model in the case of antiferromagnetic NPs. The parameters extracted from fitting are tabulated in Table 3, and two representative fitting curves are also shown in Fig. 17. From Table 3, it is evident that MF increases as wt% of NiO phase increases, and effective moment/particle also increases with a ferromagnetic contribution, consistent with the literature.^33^

**Table 3:** Results obtained from the fitting of the *M*–*H* curves

Sample name	*M*_F_ emu g^−1^	*μ*_NP_ emu g^−1^	*χ*_total_ emu g^−1^ Oe^−1^
S3	0.0043	1 × 10^−15^	0.0087
S4	0.058	1.6 × 10^−15^	0.0115
S5	0.0653	1.9 × 10^−15^	0.0216
S6	0.102	2.7 × 10^−15^	0.01462
S7	0.12	3 × 10^−15^	0.033
S8	0.13	3.3 × 10^−15^	0.0197
S9	0.18	4.3 × 10^−15^	0.036

### 3.6 Conclusive comment on the origin of magnetism in Zn(Ag, Ni)O

It is observed that low temperature magnetic ordering is observed in low Ni doped samples and shifts to high temperatures as Ni doping increases. The fraction of NiO phases also increases with an increase in doping. If hysteretic behavior is solely due to the NiO phase, then the magnetic ordering will not be shifted to a higher temperature as the particle size of NiO is almost constant. B. Balaraju *et al.*^34^ mentioned that the magnetic properties of NiO are highly dependent on particle size. Therefore, particle size dependence on magnetism due to NiO can be ruled out. However, the impurity phase NiO definitely contributes to magnetic ordering, but doping with Ni and Ag also plays a crucial role in magnetism. There are a few theoretical models in the literature to explain the magnetic ordering within the sample. The RKKY (Ruderman–Kittel–Kasuya–Yosida) model and double exchange (DE) model are very popular models.^35,36^ The RKKY model is applicable in systems where localized magnetic moments interact *via* the exchange of itinerant electrons. The DE model is applicable in systems where both localized magnetic moments and itinerant electrons coexist and interact strongly. Therefore, the presence of delocalized or free-moving electrons (itinerant electrons) throughout the crystal lattice is the main criterion for the applicability of this model. Here, the host material is ZnO, and therefore the probability of the presence of a large number of itinerant electrons is comparatively low.^37^ We believe that structural defects (vacancies, interstitials, and dislocations) are the main reason for the observed magnetic properties of the sample. Defects in diluted magnetic semiconductors (DMSs) can create localized states within the semiconductor band structure, influencing the formation and behavior of bound magnetic polarons (BMPs).^38–40^ These localized states can trap charge carriers, facilitating their interaction with nearby magnetic impurities and promoting the formation of BMPs. These percolating BMP clusters enable long-range magnetic interactions between distant regions of the material.^41–43^ As a result, the material exhibits long-range magnetic ordering, characterized by the alignment of magnetic moments over macroscopic distances.

## 4. Conclusions

The sol–gel precipitation method was used to prepare Ag-doped and Ni-co-doped ZnO NPs. XRD and XPS measurements provided a confirmation of structural alterations by the dopant and co-dopant. The NiO phase was observed with the increase of Ni content in Ag-doped ZnO. A rod-like morphology with tamarind shapes was observed. There is a reduction of SHG and THG signals with the addition of more Ni content. Their *M*–*H* curves exhibit hysteresis loops when Ni is present in the samples. The bifurcation between the FC and ZFC curves indicates a super-paramagnetic interaction between particles with a wide size distribution. The underlying reason for the linear shapes of hysteresis loops is supported by the presence of the NiO phase. The core–shell model substantiated the observation.

## Conflicts of interest

There are no conflicts to declare.

## References

[cit1] Naseer S., Aamir M., Mirza M. A., Jabeen U., Tahir R., Malghan M. N. K., Wali Q. (2022). Synthesis of Ni-Ag-ZnO solid solution nanoparticles for photoreduction and antimicrobial applications. RSC Adv..

[cit2] Lemine O. M., Modwi A., Houas A., Dai J. H., Song Y., Alshammari M., Alanzi A., Alhathlool R., Bououdina M. (2018). Room temperature ferromagnetism in Ni, Fe and Ag co-doped Cu–ZnO nanoparticles: an experimental and first principles DFT study. J. Mater. Sci.: Mater. Electron..

[cit3] Jeyachitra R., Kalpana S., Senthil T. S., Kang M. (2020). Electrical behavior and enhanced photocatalytic activity of (Ag, Ni) co-doped ZnO nanoparticles synthesized from co-precipitation technique. Water Sci. Technol..

[cit4] P Misra K., K Shukla R., Srivastava A., Srivastava A. (2009). Blueshift in optical band gap in nanocrystalline films deposited by sol-gel method. Appl. Phys. Lett..

[cit5] Chattopadhyay S., Misra K. P., Agarwala A., Shahee A., Jain S., Halder N., Rao A., Babu P. D., Saran M., Mukhopadhyay A. K. (2019). Ceram. Int..

[cit6] Goktas A., Modanlı S., Tumbul A., Kilic A. (2022). Facile synthesis and characterization of ZnO, ZnO:Co, and ZnO/ZnO:Co nano rod-like homojunction thin films: Role of crystallite/grain size and microstrain in photocatalytic performance. J. Alloys Compd..

[cit7] Khayatian A., Almasi Kashi M., Azimirad R., Safa S., Akhtarianfar Akhtarian S. F. (2016). Effect of annealing process in tuning of defects in ZnO nanorods and their application in UV photodetectors. Optik.

[cit8] Goktas A., Aslan F., Tumbul A. (2015). Nanostructured Cu-doped ZnS polycrystalline thin films produced by a wet chemical route: the influences of Cu doping and film thickness on the structural, optical and electrical properties. J. Sol-Gel Sci. Technol..

[cit9] Goktas A., Aslan F., Yeşilata B., Boz İ. (2018). Physical properties of solution processable n-type Fe and Al co-doped ZnO nanostructured thin films: Role of Al doping levels and annealing. Mater. Sci. Semicond. Process..

[cit10] Kumawat A., Chattopadhyay S., Misra K. P., Halder N., Jain S. K., Choudhary B. L. (2020). Blue-shift in the optical band gap of sol-gel derived Zn_(1-x)_Sr_x_O nanoparticles. Solid State Sci..

[cit11] Kumar N., Misra K. P., Jain S. K., Choudhary B. L. (2013). Structural and morphological properties of Ce doped ZnO. AIP Conf. Proc..

[cit12] Kumawat A., Misra K. P., Chattopadhyay S. (2022). Band Gap Engineering and Relationship with Luminescence in Rare-Earth Elements Doped ZnO: An Overview. Mater. Technol..

[cit13] Misra K. P., Jain S., Agarwala A., Chattopadhyay S., Halder N. (2020). Effective mass model supported band gap variation in cobalt-doped ZnO nanoparticles obtained by co-precipitation. Semiconductors.

[cit14] Zhang Q., Liu J.-K., Wang J.-D., Luo H.-X., Lu Y., Yang X.-H. (2014). Atmospheric Self-induction Synthesis and Enhanced Visible Light Photocatalytic Performance of Fe3+ Doped Ag-ZnO Mesocrystals. Ind. Eng. Chem. Res..

[cit15] Ahmad I., Mazhar M. E., Usmani M. N., Mehmood M., Abbas W., Akhtar N., Ahmed E. (2019). Auto-combustion synthesis of pure and Er, Dy co-doped ZnO nanomaterials for efficient methyl orange degradation using solar and visible light photocatalysis. Mater. Res. Express.

[cit16] Subash B., Krishnakumar B., Swaminathan M., Shanthi M. (2013). Highly Efficient, Solar Active, and Reusable Photocatalyst: Zr-Loaded Ag–ZnO for Reactive Red 120 Dye Degradation with Synergistic Effect and Dye-Sensitized Mechanism. Langmuir.

[cit17] Al-Harbi F. F., El Ghoul J. M. (2021). Sol–Gel Synthesis of Dy Co-Doped ZnO:V Nanoparticles for Optoelectronic Applications. Condens. Matter.

[cit18] Serier H., Toulemonde O., Bernard D., Demourgues A., Majimel J., Gaudon M. (2012). Dilute magnetic semi-conductor properties of Ga/Al/Co-codoped ZnO oxides. Mater. Res. Bull..

[cit19] Sharma A., Khangarot R. K., Chattopadhyay S., Misra K. P., Misra R. D. K., Babu P. D. (2023). Band Gap Reduction and Improved Ferromagnetic Ordering via Bound Magnetic Polarons in Zn(Al, Ce)O Nanoparticles. Mater. Technol..

[cit20] Yin Y., Sibley A., Quinton J. S., Lewis D. A., Andersson G. G. (2018). Dipole Formation at the MoO_3_/Conjugated Polymer Interface. Adv. Funct. Mater..

[cit21] Chattopadhyay S., Kumawat A., Misra K. P., Halder N., Bandyopadhyay A., Antony A., Rao A., Poornesh P., Jedryka J., Ozga K., Kucharska B., Misra R. D. K. (2021). Micro-strain administered SHG intensity enhancement by heavy Ce doping in co-precipitated ZnO nanoparticles. Mater. Sci. Eng. B.

[cit22] Cui J., Jiang J., Shi L., Zhao F., Wang D., Lin Y., Xie T. (2016). The role of Ni doping on photoelectric gas-sensing properties of ZnO nanofibers to HCHO at room-temperature. RSC Adv..

[cit23] Liu W. J., Tang X. D., Tang Z., Bai W., Tang N. Y. (2013). Oxygen Defects Mediated Magnetism of Ni Doped ZnO. Adv. Condens. Matter Phys..

[cit24] Wang W., Hui S., Zhang F., Wang X., Zhang S., Yan J., Zhang W. (2019). Fabrication and Study on Magnetic-Optical Properties of Ni-Doped ZnO Nanorod Arrays. Micromachines.

[cit25] Larciprete M. C., Centini M. (2015). Second harmonic generation from ZnO films and nanostructures. Appl. Phys. Rev..

[cit26] Moura K., Lima R., Coelho A., Souza-Junior E., Duque J., Meneses C. (2014). Tuning the surface anisotropy in Fe-doped NiO nanoparticles. Nanoscale.

[cit27] Sarkar B. J., Bandyopadhyay A. (2021). Studies of magnetic behavior of chemically synthesized interacting superparamagnetic copper ferrite nanoparticles. J. Mater. Sci.: Mater. Electron..

[cit28] Sarkar B. J., Bandyopadhyay A. (2022). Quantitative analysis of the magnetic properties of a mixture of single- and multi-domain Zn-substituted CuFe2O4 nanoparticles with canted spin. J. Mater. Sci.: Mater. Electron..

[cit29] Mandal S., Banerjee S., Menon K. S. R. (2009). Core-shell model of the vacancy concentration and magnetic behavior for antiferromagnetic nanoparticle. Phys. Rev. B: Condens. Matter Mater. Phys..

[cit30] Mandal S., Menon K. S. R., Mahatha S. K., Banerjee S. (2011). Finite size versus surface effects on magnetic properties of antiferromagnetic particles. Appl. Phys. Lett..

[cit31] Balaev D. A., Krasikov A. A., Popkov S. I., Semenov S. V., Volochaev M. N., Velikanov D. A., Kirillov V. L., Martyanov O. N. (2021). Uncompensated magnetic moment and surface and size effects in few-nanometer antiferromagnetic NiO particles. J. Magn. Magn. Mater..

[cit32] De Biasi E., Ramos C. A., Zysler R. D., Romero H. (2002). Large surface magnetic contribution in amorphous ferromagnetic nanoparticles. Phys. Rev. B: Condens. Matter Mater. Phys..

[cit33] Bandyopadhyay A., Deb A. K., Kobayashi S., Yoshimura K., Chakrabarti P. K. (2014). Room temperature ferromagnetism in Fe-doped europium oxide (Eu_1.90_Fe_0.10_O_3−δ_). J. Alloys Compd..

[cit34] Balaraju B., Kaleemulla S., Rao N. M., Omkaram I., Reddy D. S., Subbaravamma K., Rao G. V. (2018). Effect of Fe Substitution on Microstructure and Magnetic Properties of Ni_1−x_Fe_x_O_2_ Nanoparticles. J. Supercond. Nov. Magnetism.

[cit35] Yosida K. (1957). Magnetic Properties of Cu-Mn Alloys. Phys. Rev..

[cit36] Anderson P. W., Hasegawa H. (1955). Considerations on Double Exchange. Phys. Rev..

[cit37] Bandyopadhyay A., Bhakta N., Sutradhar S., Sarkar B. J., K Deb A., Kobayashi S., Yoshimura K., Chakrabarty P. K. (2016). Microstructure investigation, optical properties and magnetic phase transition of Tm^3+^ substituted nanocrystalline ZnO (Zn_0.95_Tm_0.05_O). RSC Adv..

[cit38] Goktas S., Tumbul A., Goktas A. (2023). Growth Technique–Induced Highly C-Axis-Oriented ZnO: Mn, Zno: Fe and ZnO: Co Thin Films: A Comparison of Nanostructure, Surface Morphology, Optical Band Gap, and Room Temperature Ferromagnetism. J. Supercond. Nov. Magnetism.

[cit39] Kocyigit A., Topkaya R. (2019). Structural, optical and magnetic properties of Ni-Co co-doped ZnO thin films. Mater. Res. Express.

[cit40] Aba Z., Goktas A., Kilic A. (2024). Characterization of Zn_1-x_La_x_S thin films; compositional, surface, optical, and photoluminescence properties for possible optoelectronic and photocatalytic applications. J. Sol-Gel Sci. Technol..

[cit41] Aslan E., Sahin G., Goktas A. (2023). Facile synthesis of Sb_2_S_3_ micro-materials for highly sensitive visible light photodetectors and photocatalytic applications. Mater. Chem. Phys..

[cit42] Aslan E., Emir Ö., Arslan F., Goktas A., Tumbul A., Durgun M., Kilic A., Aktacir M. A., Aslan F. (2024). Improving the optical properties of CuCoMnO_x_ spinel absorber using ZnO nanorod arrays for thermal collector and photocatalytic applications. Ceram. Int..

[cit43] Aslan F., Arslan F., Tumbul A., Goktas A. (2022). Synthesis and characterization of solution processed p-SnS and n-SnS_2_ thin films: Effect of starting chemicals. Opt. Mater..

